# Valerian Inhibits Rat Hepatocarcinogenesis by Activating GABA(A) Receptor-Mediated Signaling

**DOI:** 10.1371/journal.pone.0113610

**Published:** 2014-11-24

**Authors:** Anna Kakehashi, Ayumi Kato, Naomi Ishii, Min Wei, Keiichirou Morimura, Shoji Fukushima, Hideki Wanibuchi

**Affiliations:** Department of Molecular Pathology, Osaka City University Graduate School of Medicine, Osaka, Japan; Wayne State University School of Medicine, United States of America

## Abstract

Valerian is widely used as a traditional medicine to improve the quality of sleep due to interaction of several active components with the γ-aminobutyric acid (GABA) A receptor (GABA(A)R) system. Recently, activation of GABA signaling in stem cells has been reported to suppress cell cycle progression *in vivo*. Furthermore, possible inhibitory effects of GABA(A)R agonists on hepatocarcinogenesis have been reported. The present study was performed to investigate modulating effects of Valerian on hepatocarcinogenesis using a medium-term rat liver bioassay. Male F344 rats were treated with one of the most powerful Valerian species (Valeriana *sitchensis*) at doses of 0, 50, 500 and 5000 ppm in their drinking water after initiation of hepatocarcinogenesis with diethylnitrosamine (DEN). Formation of glutathione S-transferase placental form positive (GST-P^+^) foci was significantly inhibited by Valerian at all applied doses compared with DEN initiation control rats. Generation of 8-hydroxy-2′-deoxyguanosine in the rat liver was significantly suppressed by all doses of Valerian, likely due to suppression of Nrf2, CYP7A1 and induction of catalase expression. Cell proliferation was significantly inhibited, while apoptosis was induced in areas of GST-P^+^ foci of Valerian groups associated with suppression of c-myc, Mafb, cyclin D1 and induction of p21^Waf1/Cip1^, p53 and Bax mRNA expression. Interestingly, expression of the GABA(A)R alpha 1 subunit was observed in GST-P^+^ foci of DEN control rats, with significant elevation associated with Valerian treatment. These results indicate that Valerian exhibits inhibitory effects on rat hepatocarcinogenesis by inhibiting oxidative DNA damage, suppressing cell proliferation and inducing apoptosis in GST-P^+^ foci by activating GABA(A)R-mediated signaling.

## Introduction

Valerian root extract, widely used in Europe and America as a sedative, hypnotic and anxiolytic, contains a variety of constituents, including essential oils that appear to contribute to the sedating properties of the herb. Iridoid valepotriates like bornyl isovalerenate and bornyl acetate, valeric, isovaleric, formic, malic and acetoxyvalerenic acids, alkaloids and lignans are among components with possible benefit [1]. Some of these are known to bind to GABA(A)Rs to exert sedating effects [1]. Valerian extracts have been demonstrated to exert a variety of effects on GABAergic neurons in laboratory animals, including increased release of GABA, decreased GABA reuptake, and decreased GABA degradation [2].

Valerian effects on the central nervous system (CNS) are thought to be similar to those of pharmaceutical phenobarbital (PB), a sedative and anticolvulsant which also binds to GABA(A)Rs and is used widely in clinical therapy for long-term treatment [3]. Our previous research indicated that formation of rat liver preneoplastic lesions, GST-P^+^ foci, and liver tumors induced by the genotoxic hepatocarcinogen, diethylnitrosamine, was inhibited at low doses (1 and 2 ppm) in a rat liver medium-term bioassay [4], and after 10 and 33 weeks of PB administration in a 2-step liver carcinogenesis model [5]. The mechanism of suppression of GST-P^+^ foci and tumor development by low doses of PB was suggested to be related to inhibitory effects on cellular proliferation within the areas of preneoplastic lesions, and a correlation was suggested with overexpression of GABA producing enzyme glutamic acid decarboxylase 65 [5]. Furthermore, a negative correlation between expression of GABA(A)Rs in hepatocytes and thymidine incorporation in liver specimens has recently been reported, albeit without evidence of a causal relationship, and GABA A and B receptor subtypes appear to contribute to hepatocyte DNA synthesis, mediation of growth stimulation and suppression of cell proliferation in the rat liver through regulation of sympathetic activity [6],[7],[8]. Moreover, GABA(A)R-mediated signaling was recently shown to cause S-phase cell cycle arrest in embryonic stem and neural crest stem cells by promoting phosphorylation of histone H2AX [9]. These results support the idea that Valerian may exert an inhibitory effect on development of preneoplastic and neoplastic liver lesions.

To check this hypothesis, in the present study we employed a medium-term rat liver bioassay which has been shown to be a very useful tool for detection of hepatocarcinogenicity and chemopreventive potential of chemicals [10], to investigate the modifying effects of water root extract of one of the most powerful Valerian species, Valeriana *sitchensis*, containing high levels of valepotriates. The effects of Valerian at doses of 50, 500 and 5000 ppm applied in the drinking water were investigated in rats with reference to preneoplastic lesion development, oxidative stress, DNA damage, cellular proliferation, apoptosis and gene expression changes in the liver.

## Materials and Methods

### Chemicals

DEN was from Sakai Research Laboratory (Fukui, Japan). All other reagents were purchased from Sigma-Aldrich or Wako Pure Chemicals Industries (Osaka, Japan).

### Valeriana sitchensis root extract

Valerian root extracts usually contain more than 100 different constituents. The Valeriana *sitchensis* alcohol-free extract used in the present study was obtained from Eclectic Institute Inc. (Oregon, USA). It is made first using organic sugar cane alcohol, which is then removed using the Lloyd Extractor. After the alcohol has been removed, the glycerin is added. Glycerin is a solvent similar to alcohol and is listed in the US Pharmacopoeia as an agent to administer certain constituents. Present Valerian extract contained iridoid valepotriates: valtrate, valtrate isovaleroxyhydrin, acevaltrate, valechlorine, didrovaltrate, homodidrovaltrate, deoxydodidrovaltrate, isovaleroxyhydroxydidrovaltrate, isovaltrate, 7-epi-deacetyl-isovaltrate. In addition, it contains valerosidatum (an iridoid ester glycoside). Volatile oil contains a lot of components including valeric and isovaleric acids, bornyl acetate, monoterpens (e.g. α and β-pinene, camphene, borneol, isoeugenyl isovalerate, eugenyl isovalerate), sesquiterpenes β-bisabolene, caryophyllene, valeranone, valerenic acid (traces), valerianol, valerenal,β-ionone, patchouli alcohol, ledole and terpinolene among others. Furthermore, extract includes alkaloids (e.g. actinidine, valerianine, valerin, chatinin), choline, methyl 2-pyrrolyl ketone, chlorogenic acid, caffeic acid, β-sitosterol, tannin, gam, manganese, calcium, amino acids such as GABA, glutamine, arginine, alanine and others. Highest amounts of valerenic acid were reported in V. *officinalis* L., trace amounts in V. *sitchensis*, and none in the other Valerian species analyzed [11].

### Animals and treatment

A total of 120, five-week-old male Fisher 344 rats (Charles River, Japan, Hino, Shiga, Japan) were quarantined for 1 week before the start of the experiment. They were housed in an animal facility maintained on a 12 h (7:00–19:00) light/dark cycle, at a constant temperature of 23±1°C and relative humidity of 44±5%, and given free access to tap water and food (Oriental MF pellet diet, Oriental Yeast Co., Tokyo, Japan). All experimental procedures were conducted following approval of the Animal Care and Use Committee of the Osaka City University Graduate School of Medicine. Guidelines set by the National Institute of Health and Public Health Service Policy on the Humane Use and Care of Laboratory Animals were followed at all times.

Before the start of the experiment, 6-week-old rats were allocated to six groups (groups 1–4, 25 rats/group; groups 5 and 6, 10 rats/group). In groups 1–4, rats were given a single i.p. injection of DEN (200 mg/kg body weight (b.w.)) dissolved in saline to initiate hepatocarcinogenesis. In groups 5 and 6, rats were administered an i.p. injection of saline as vehicle controls. After 2 weeks on tap water and basal diet, animals in groups 1–4 were administered water root extract of Valeriana *sitchensis* (Valerian) in their drinking water at doses of 0, 50, 500 and 5000 ppm, for 6 weeks from weeks 3 to 8. All were subjected to two thirds partial hepatectomy (PH) at week 3 to maximize any interaction between proliferation and the effects of the test chemicals. At sacrifice at week 8, livers were quickly dissected out, weighed and sections from three lobes were fixed in Bouin's solution and 10% phosphate-buffered formalin for histological and immunohistochemical analyses. In addition, samples were frozen in liquid nitrogen and stored at −80°C for molecular analyses. For AST and ALT measurements by the consensus method of Japan Society of Clinical Chemistry, blood was collected from the abdominal aorta.

### Selection of the doses

Valerian doses used in the present study were selected on the basis of previously published data on humans and the findings of our preliminary experiment in which no toxicity was detected even at a dose of 5000 ppm. The doses of 50 ppm (5 mg/kg b.w./day), 500 ppm (50 mg/kg b.w./day) and 5000 ppm (500 mg/kg b.w./day) consumed by a rat (b.w. 200 g) in 20 ml drinking water in the present experiment would be equal to 0.05, 0.5 and 5 mg/kg b.w./day intake by a human with a mean body weight of 50 kg (the accepted WHO safety factor in terms of accepted dietary intake (ADI) for rats is 100). Another extrapolation from human to rat involves multiplying the human dose by 6.16 (Km human/Km animal = 37/6) [12]. In this case, the animal doses of 5, 50 and 500 mg/kg b.w./day would be equal to 0.8, 8.1, and 81.2 mg/kg b.w./day intake, respectively, by humans.

### Immunohistochemical analyses

Immunohistochemical assessment of GST-P was performed with the ABC method as described by Kitano *et al.* [4] using rabbit polyclonal GST-P (IgG, MBL Co., Nagoya, Japan, 1∶2000) antibody. Quantitation of GST-P^+^ foci was accomplished using two-dimensional evaluation [13]. The numbers and areas of foci greater than 0.2 mm in diameter, and total areas of liver sections, were measured using a color image processor (IPAP; Sumica Technos Osaka, Japan) to give values per cm^2^ of liver section.

Double stainings for GST-P and PCNA and GST-P and TUNEL were performed in formalin-fixed sections with polyclonal rabbit anti-GST-P antibody at 1∶2000 dilution, anti-PCNA mouse monoclonal (PC-10, IgG2a; DAKO, Kyoto, Japan; 1∶500) antibody and ApopTaq Peroxidase in Situ Apoptosis Detection Kit (Intergen Co., Purchase, NY) using alkaline phosphatase (Vectastain ABC-AP kit, Vector Red) solution for the immunohistochemical detection of GST-P and DAB for the detection of PCNA or apoptosis [5]. The labeling indices were calculated in the area of GST-P^+^ foci and background liver parenchyma (5000 cells) and expressed as percentages of positive cells for PCNA or ssDNA in all GST-P^+^ cells or surrounding liver cells.

Liver sections of selected animals were stained immunohistochemically using rabbit polyclonal GABA(A)RA1 (Abcam, 1∶300), mouse monoclonal GABA(B)R1 (Abcam, 1∶500), rabbit polyclonal GABA(B)R2 (Abcam, 1∶100) and anti-phospho-Nrf2 (Ser40) (Nrf2-Ser-P; Bioss, 1∶200) antibodies by ABC method, with color development by DAB, and assessed qualitatively [14]. Furthermore, double staining for GABA(A)RA1 and PCNA was performed using alkaline phosphatase (Vectastain ABC-AP kit, Vector blue) and DAB for the detection of GABA(A)RA1 and PCNA, respectively. Negative controls were included in every staining and immunostained as described above, but with primary serum instead of antibodies.

### 8-OHdG analysis

DNA samples were extracted from rat liver tissues to allow measurement of 8-OHdG levels by HPLC-ECD as reported previously [15].

### cDNA microarray analysis

Total RNA was isolated from rat liver tissues and 8 µg pooled aliquots from 5 rats in each group were treated with DNase 1 and processed for PolyA^+^ RNA enrichment and generation of cDNA probes using a Affymetrix GeneChip T7-Oligo(dT) Promoter Primer Kit (Affymetrix) according to the manufacturer's protocol. Biotin-labeled antisense cRNA was synthesized by *in vitro* transcription reaction using an RNA Transcript Labeling Kit (Affymetrix, P/N 900182), purified and fragmented, and hybridized to GeneChip RAT Genome 230 2.0 arrays, with 28,700 probe sets. Affymetrix GCOS software version 1.0 was employed for normalization and for monitoring specific hybridization. Microarray data were analyzed using GeneSpring software version 12 (Agilent Technologies, 1998-2012, Silicon Genetics, Redwood City, CA). Each array was normalized to the 50^th^ percentile and each gene was normalized to the control (DEN initiated rats). Microarray analysis was repeated three times to check the reproducibility of the data and mean values of gene expression were calculated for spots with at least 2 fold up- or down-regulation. One-way-Anova was applied to compare replicate mean values of control and experimental groups and to find genes whose expression was consistently altered by Valerian administration. Clustering analysis was performed with the Condition Tree algorithm. The dataset was submitted to DNA Data Bank of Japan (DDBJ) (submission ID: PSUB003813).

To assign biological significance of differentially expressed genes and identify networks of interacting genes, functional groups and pathways, the Ingenuity program (Ingenuity Systems, Mountain View, CA) was utilized. IPA was further applied for the prediction of altered up-stream regulators by Valerian. Transcriptional regulation was measured by z-scores. A z-score of above 2 was considered significant.

### Real-time quantitative reverse transcription-PCR (Q-RT-PCR)

Real-time Q-PCR was performed as previously described [16] using TaqMan probes and primer sets from TaqMan Gene Expression Assays (Applied Biosystems, Japan) for the analysis of mRNA expression of *GABA(A)RA1* (Rn00788315_m1), *histone deacetylase 4* (*HDAC4*) (Rn01427040_m1), *c-myc* (Rn00561507_m1), *mafb* (Rn00709456_s1), *jun* (Rn00572991_m1), *fos* (Rn02396760_g1), *MAPK 3* (*ERK1*)(Rn00820922_g1), *MAPK 14* (*p38*) (Rn00578842), *nuclear factor (erythroid-derived 2)-like 2* (*Nrf2*) (Rn00477784_m1), *NADPH quinone oxidoreductase 1* (*NQO1*) (Rn01432447_m1), *heme oxygenase 1* (*HO-1*) (Rn00561387_m1), *cyp7A1* (Rn00564065_m1), *superoxide dismutase* (*SOD*) (Rn00566938_m1), *catalase* (*CAT*) (Rn00560930_m1), *cyclin D1* (Rn00432359_m1), *NfkB* (Rn01502266_m1), *p53* (RN00755717_m1), *p21 ^Waf1/cip1^* (Rn01427989_s1) and *Bax* (Rn02532082_g1). Results are expressed relative to the number of eukaryotic *18S* RNA transcripts (4319413E) used as an internal control.

### Statistical analysis

The significance of differences for each parameter (excluding general conditions) was analyzed using the StatLight–2000(C) program (Yukms corp., Japan) or the IBM SPSS Statistics 19 Software (IBM, USA). Statistical comparisons between groups of numerical data were conducted using the Bartlett's test. If homogeneous, the data were analyzed with the Dunnett's multiple comparison test (two sided), and if not, with the Steel's test (two-sided). Statistical comparisons between vehicle control and 5000 ppm Valerian groups for numerical data were assessed using the F test. If homogeneous, the data were analyzed with the Student's t-test (two-sided), and if not, with the Welch test. In microarray analysis, GeneSpring software version 12 was utilized to perform one-way-Anova to compare replicate mean values of control and experimental groups and to find genes whose expression was consistently altered by Valerian administration.

## Results

### General observations

Final body and relative liver and kidney weights and Valerian intake data are shown in Table 1. There were no significant differences among the groups with regard to water and food consumption or body weight gain. No changes in liver or kidney/body weight ratios were induced by administration of Valerian at any doses as compared to the initiation control or vehicle control groups. Valerian administration after DEN initiation resulted in suppression of AST levels in the blood serum in a dose-dependent manner as compared with the initiation control values which were significantly higher than in vehicle control group (Table 1). No influence of DEN or Valerian application was noted on serum levels of ALT in all groups. Several animals died by 2 days after the PH (DEN initiation control group (1 rat), DEN→Val, 50 ppm group (1 rat), DEN→Val, 500 ppm group (3 rats) DEN→Val, 5000 ppm group (2 rats) and vehicle control group (1 rat)).

**Table 1:** Body, relative liver and kidney weights and AST and ALT levels in the blood serum of rats treated with DEN or vehicle (saline) and administered Valerian at doses of 50, 500 and 5000 ppm.

Group	No. rats	Body weight (g)	Valerian intake (mg/kg/day)	Relative liver weight (g)	Relative kidneys weight (g)	AST(IU/L)^d^	ALT (IU/L)^d^
DEN	24	270±11	0	3.08±0.14	0.63±0.02	115.0±6.4	53.7±5.1
DEN→ Val,50 ppm	24	268±14	5.34	3.06±0.10	0.63±0.02	106.0±10.4	52.9±6.1
DEN→ Val,500 ppm	22	271±14	49.83	3.13±0.16	0.61±0.03	104.7±14.8^a^	57.3±11.8
DEN→ Val,5000 ppm	23	272±10	501.16	3.15±0.14	0.63±0.03	96.8±6.3^b^	55.5±7.2
Vehicle	9	287 ±10	0	3.19 ±0.13	0.63 ±0.02	87.4±7.9^c^	52.2±7.5
Vehicle→ Val,5000 ppm	10	284±19	517.93	3.16 ±0.24	0.63 ±0.03	90.1±13.6	56.9±10.5

Data are Mean ± SD; ^a^P<0.05; ^b^P<0.01; ^c^P<0.0001 vs DEN control group ^d^(n = 10) for DEN, DEN→Val, 50 ppm, DEN→Val, 500 ppm, DEN→Val, 5000 ppm, Vehicle→Val,5000 ppm groups and (n = 9) for Vehicle control group.

### Inhibitory effects of Valerian on development GST-P^+^ foci

**Figure 1:**
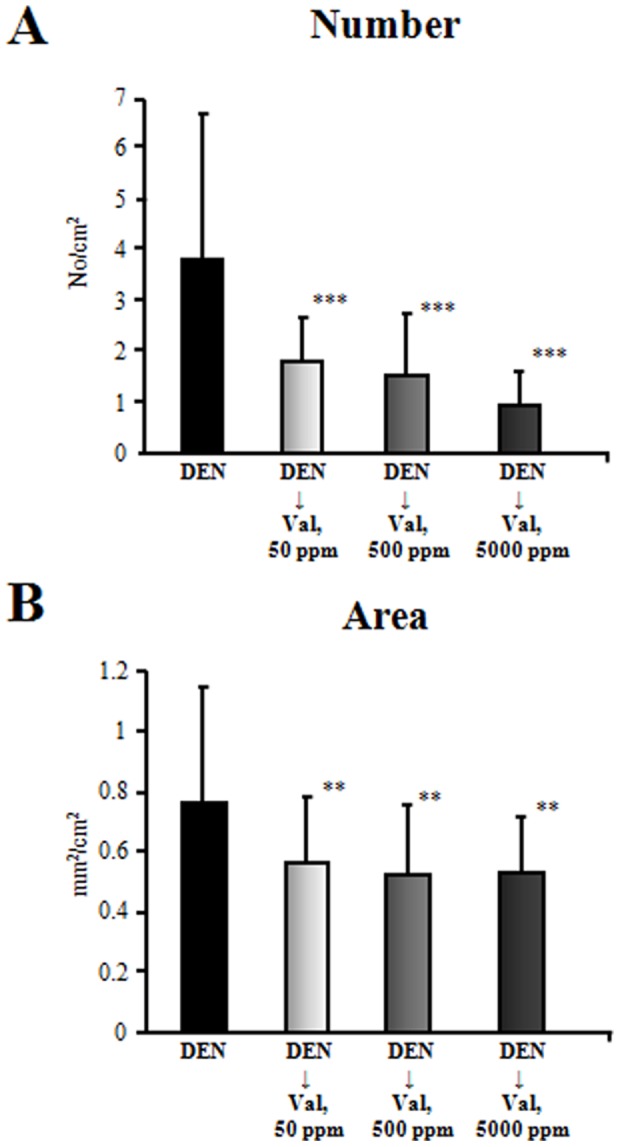
Numbers and areas of GST-P^+^ foci in the livers of rats treated with DEN or vehicle and administered Valerian at doses of 50, 500 and 5000 ppm. Data are Mean ± SD (all survived rats were subjected to analysis). ^**^P<0.01; ^***^P<0.0001 versus the DEN control group.

Data for GST-P^+^ foci are given in Figure 1A and B. No GST-P^+^ foci were detected in vehicle control and 5000 ppm Valerian treated rats. Importantly, both numbers and to a lesser extent areas of GST-P^+^ foci in rats given 50, 500 and 5000 ppm Valerian after DEN initiation were significantly decreased in a dose-dependent manner as compared to the initiation control group.

### Alteration in cell proliferation

**Figure 2:**
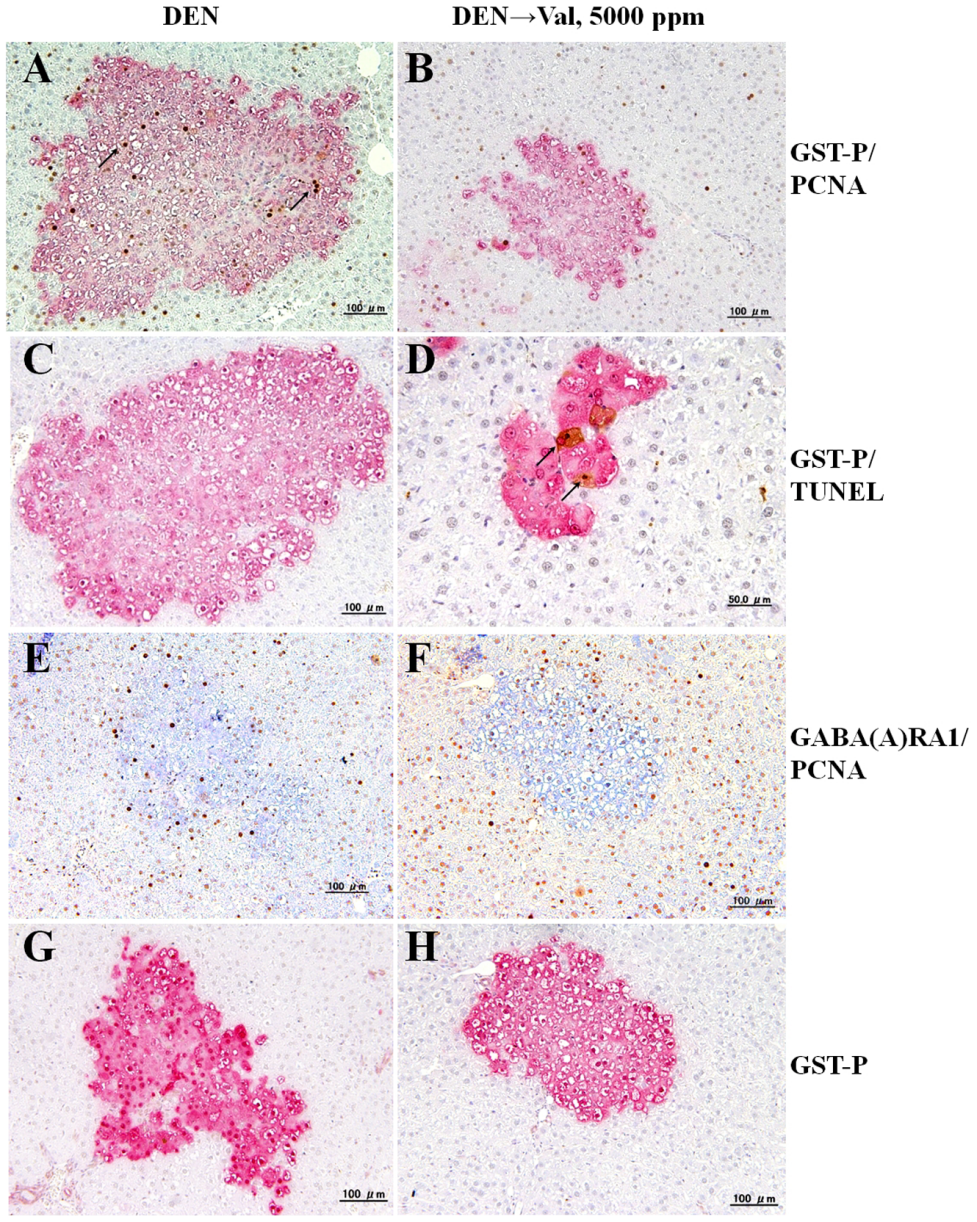
Double immunohistochemistry for GST-P (red) and PCNA (brown/black) (A, B), GST-P (red) and apoptosis (TUNEL) (black) (C, D), and immunohistochemical assessment in serial sections of GABA(A)RA1 (blue) and PCNA (brown/black) (E, F) and GST-P (red) (G, H) in the livers of F344 rats treated with DEN (A, C, E, G) or Valerian at a dose of 5000 ppm after DEN initiation (B, D, F, H). E and G, F and H are serial sections, respectively. Three preparations of liver tissue for each rat were used in the analysis of GABA(A)RA1^+^/GST-P^+^ foci numbers. 5000 cells on the normal-appearing liver tissue and total cells in the GST-P^+^ foci areas were checked for PCNA and apoptotic indices. Note the decreased number of PCNA positive cells and induction of apoptosis in GST-P^+^ foci in 5000 ppm Valerian treated rats, with membranous overexpression of GABA(A)RA1 in GST-P^+^ foci after DEN initiation.

Representative double staining pictures of GST-P and PCNA in rats administered DEN followed with 5000 ppm Valerian and DEN alone are shown in Figure 2. Different patterns of PCNA staining are believed to correlate with the individual phases of the cell cycle (Gl, S, G2, M, G_0_). In the initiation control group several hepatic foci, consisting of more than 20 cells, contained a large number of PCNA positive nuclei (Fig.2A). Dose-dependent decreases of PCNA indices as compared to the respective controls were obvious in the areas of GST-P^+^ foci and surrounding liver tissue, with significant difference observed for GST-P^+^ foci in 500 and 5000 ppm Valerian administered rats after DEN initiation (Table 2 and Fig.2A and B). In surrounding liver tissue significantly suppressed PCNA values were found in rats treated with 5000 ppm Valerian alone as compared to vehicle control group.

**Table 2:** Alteration of cell proliferation and apoptosis in GST-P^+^ foci and surrounding liver tissue, 8-OHdG formation and GABA(A)RA1/GST-P^+^ foci numbers in the livers of rats treated with DEN or vehicle (saline) and administered Valerian at doses of 50, 500 and 5000 ppm.

Group	No.rats	PCNA (%)		Apoptosis (%)		GABA(A)RA1^+^/GST-P^+^ foci (No./cm2)	8-OHdG (8-OHdG/10^5^dG)^e^
		GST-P^+^ foci	Surrounding area	GST-P^+^ foci	Surrounding area		
DEN	24	16.40±8.21	13.63±8.21	0.06±0.08	0.13±0.06	0.24±0.35	0.31±0.06
DEN→ Val,50 ppm	24	14.59±4.48	13.30±1.64	0.14±0.14	0.11±0.07	0.58±0.58	0.26±0.04^a^
DEN→ Val,500 ppm	22	8.38±3.09^b^	11.91±3.65	0.45±0.30^c^	0.27±0.11^b^	0.63±0.62	0.26±0.04^a^
DEN→ Val,5000 ppm	23	6.73±1.55^c^	11.58±1.43	0.68±0.39^d^	0.32±0.14^c^	0.96±0.79^b^	0.25±0.03^a^
Vehicle	9	ND	11.16±0.95	ND	0.30±0.04^b^	ND	0.25±0.03^a^
Vehicle→ Val,5000 ppm	10	ND	9.73±0.64*	ND	0.39±0.03**	ND	0.23±0.03*

Data are Mean ± SD; ^a^P<0.05; ^b^P<0.01; ^c^P<0.001; ^d^P<0.0001 vs DEN control group; ^e^(n = 10) for DEN, DEN→Val, 50 ppm, DEN→Val, 500 ppm, DEN→Val, 5000 ppm, Vehicle→Val, 5000 ppm groups and (n = 9) for Vehicle control group. *P<0.05 and **P<0.01 vs vehicle control group; ND: not detected.

### Evaluation of apoptosis

Analysis by double immunohistochemistry for GST-P and TUNEL demonstrated dose-dependent increase of apoptosis in the areas of GST-P^+^ foci and surrounding liver tissue in the livers of rats treated with DEN followed by Valerian at doses of 500 and 5000 ppm (Table 2 and Fig.2C and D). This increase was more pronounced in the GST-P^+^ foci (4.7-fold). Slight but significant elevation of apoptosis was also apparent in the liver of rats treated with 5000 ppm Valerian alone as compared to the respective vehicle control.

### Overexpression of GABA(A)RA1 in GST-P ^+^ foci

Interestingly, a few GST-P^+^ foci in initiation control rats were positive for GABA(A)RA1 with a membranous and/or rarely cytoplasmic localization (Fig.2E and G). Furthermore, its expression was elevated in the GST-P^+^ foci of Valerian administered rats (Fig.2F and H). The numbers of GABA(A)RA1^+^/GST-P^+^ foci were dose-dependently increased in the liver of Valerian treated rats as compared to the DEN control group with significant difference observed for the high dose Valerian group (Table 2). Very few PCNA positive nuclei were observed in GABA(A)RA1^+^/GST-P^+^ foci (Fig.2F). We did not observe GABA(B)R1 or GABA(B)R2-positive cells in areas of GST-P^+^ foci of either DEN control or Valerian administered animals.

### Influence on 8-OHdG formation and Nrf2-Ser-P expression

HPLC analysis demonstrated 8-OHdG levels in liver DNA to be significantly decreased in all groups administered Valerian after DEN initiation (Table 2) demonstrating a similar pattern to those for GST-P^+^ foci numbers, areas, alteration in blood AST levels and cell proliferation in GST-P^+^ foci (Tables 1 and 2). Formation of oxidative base modifications was increased in the DEN control as compared to the vehicle control group (Table 2).

Immunohistochemical analysis of phosphorylated (activated) form of indicator of oxidative stress, Nrf2 (Nrf2-Ser-P), have demonstrated that its expression is high mostly in the areas of central and portal veins and inflammatory cell infiltration areas in the livers of rats of DEN initiation control group, but is suppressed by Valerian treatment (Figure S1).

### Alterations of gene expression induced by Valerian

The results of cDNA microarray, GeneSpring and one-way-Anova analyses of differentially expressed genes after 6 weeks of 50, 500 and 5000 ppm Valerian administration followed DEN initiation are presented in Table 3. In all livers of Valerian treated rats, we observed that many genes related to cell proliferation were down-regulated, while genes involved in apoptosis were induced as compared to the initiation control group (Table 3). Thus, dose-dependent suppression of mRNA expression of some genes upregulated in the livers of animals in the DEN control group was detected in the livers of rats administered 50, 500 and 5000 ppm Valerian after initiation. Those included c-myc and Mafb, period homolog 2 (Per2) and nuclear receptor subfamily 0, group B, member 2 (Nr0b2) transcriptional factors, insulin-like growth factor binding protein 1 (Igfbp1), serum/glucocorticoid-regulated (Sgk) and SNF1-like (Snf1lk) kinases, HIF-1 responsive RTP801 (Rtp801), protein tyrosine phosphatase, non-receptor type 16 (Ptpn16) and cyclin D1 (CD1). Furthermore, suppression of cytochrome P450, family 7, subfamily A, polypeptide 1 (Cyp7A1) catalyzing a rate-limiting step in cholesterol catabolism and bile acid biosynthesis and glutathione S-transferase, pi 2 (Gstp2) by Valerian was found. Furthermore, Valerian application induced elevation of mRNA expression of some genes whose expression was suppressed in the initiation control group, including cellular tumor antigen p53 (p53), cyclin-dependent kinase inhibitor 1A (p21^WAF1/Cip1^) and inositol 1,4,5-trisphosphate receptor, type 1 (Itpr1) and transcriptional regulators such as hepatocyte nuclear factor 3 beta, Kruppel-like factor 9 and early growth response 1.

**Table 3:** Differentially expressed genes in the livers of rats treated with DEN and administered Valerian at doses of 50, 500 and 5000 ppm, identified by Affimetrix microarray analysis.

Description	Function	Reference sequence	DEN vs Vehicle	DEN→Val, 50 ppm vs Vehicle	DEN→Val, 500 ppm vs Vehicle	DEN→ Val, 5000 ppm vs Vehicle	One-way Anova (*P* value)
V-myc avian myelocytomatosis viral oncogene homolog (c-Myc)	TR,CP,CC	NM_012603	2.50	2.98	1.03	0.78	0.049
V-maf musculoaponeurotic fibrosarcoma oncogene family, protein B (avian) (Mafb)	TR,BS	U56241	2.33	3.03	1.68	0.82	0.020
Period homolog 2 (Per2)	TR	NM_031678	2.67	1.60	1.20	0.96	0.033
Nuclear receptor subfamily 0, group B, member 2 (Nr0b2)	TR,LM	NM_057133	4.22	4.86	4.09	2.19	0.014
Hepatocyte nuclear factor 3, beta (Hnf3b)	TR,ST	NM_012743	0.25	0.25	0.56	0.65	0.020
Kruppel-like factor 9 (Klf9)	TR,ST	NM_057211	0.48	0.91	1.03	0.95	0.027
Early growth response 1 (Egr1)	TR	NM_012551	0.47	0.98	1.68	1.34	0.033
Zinc finger protein 354A (Znf354a)	TR	NM_052798	1.00	2.51	2.67	1.00	0.020
Ubiquitin specific protease 2 (Usp2)	TR	NM_053774	0.85	1.93	1.79	1.28	0.019
Insulin-like growth factor binding protein 1(Igfbp1)	CP,CM	NM_013144	11.39	6.26	3.30	2.16	0.014
Serum/glucocorticoid regulated kinase (Sgk)	CP,CM	NM_019232	4.25	1.62	1.62	1.19	0.026
SNF1-like kinase (Snf1lk)	CC,D	NM_021693	2.70	1.54	1.03	1.03	0.014
HIF-1 responsive RTP801 (Rtp801)	CP,CG	NM_080906	2.66	2.37	1.94	1.54	0.014
Protein tyrosine phosphatase, non-receptor type 16 (Ptpn16)	CC	NM_053769	2.37	1.87	0.95	0.73	0.025
Inhibitor of DNA binding 4 (Idb4)	CP,CC	NM_175582	2.25	1.40	1.37	0.95	0.030
Cyclin D1 (CD1)	CP,CC	NM_171992	2.00	1.36	1.18	1.00	0.030
Cyclin-dependent kinase inhibitor 1A (p21^WAF1/Cip1^)(CDKN1A)	CC,A	U24174	0.79	1.37	1.42	1.84	0.030
Cellular tumor antigen TP53 (p53)	CC,A	P04637	0.37	1.60	1.69	1.82	0.048
Cytochrome P450, family 7, subfamily A, polypeptide 1 (Cyp7A1)	LM, BAB	NM_012942	4.48	4.17	4.12	1.75	0.019
Glucose-6-phosphatase, catalytic (G6pc)	M	U07993	3.10	2.05	1.40	1.30	0.029
Tyrosine aminotransferase (Tat)	M	M18340	2.36	2.15	1.91	1.13	0.033
Aminolevulinic acid synthase 1 (Alas1)	M	NM_024484	2.17	2.58	2.37	2.26	0.037
Glutathione-S-transferase, alpha type2 (Gsta2)	XM	M25891	3.48	5.60	4.28	3.48	0.018
Glutathione S-transferase, pi 2 (Gstp2)	XM	X02904	2.44	3.71	2.46	1.93	0.042
Peroxisomal Ca-dependent solute carrier-like protein (Pcscl)	T	NM_145677	2.41	2.41	1.06	0.70	0.014
Solute carrier family 34 (sodium phosphate), member 2 (Slc34a2)	T	NM_053380	2.66	1.14	0.88	0.80	0.019
Solute carrier family 38, member 2 (Slc38a2)	T	AF249673	2.40	2.23	1.78	0.96	0.019
Probasin (LOC54193)	T	NM_019125	5.10	9.59	8.98	8.21	0.009
Glucocorticoid-induced leucine zipper (Gilz)	T	NM_031345	2.02	2.44	2.22	2.00	0.028
Inositol 1,4,5-triphosphate receptor 1 (Itpr1)	T	J05510	2.19	5.65	4.62	4.73	0.042
ATP synthase, H+ transporting, mitochondrial F1F0 complex, subunit e (Atp5k)	T,M	BI291386	0.43	0.43	0.89	0.92	0.048
Regulator of G-protein signaling 3 (Rgs3)	ST	NM_019340	0.26	0.43	0.54	0.59	0.033
Dual specificity phosphatase 6 (Dusp6)	ST,IM	NM_053883	0.42	0.60	0.94	1.19	0.041
Cytokine inducible SH2-containing protein (Cish)	ST	AF065161	0.19	0.53	0.33	0.49	0.019
Prolactin receptor (Prlr)	R	M57668	2.14	1.09	1.75	1.07	0.046
Interferon-related developmental regulator 1 (Ifrd1)	D	NM_019242	2.33	4.64	2.49	1.12	0.044
ATPase inhibitor (Atpi)	ANG	NM_012915	0.46	0.47	0.61	0.71	0.037

TR: transcription regulation; CP: cell proliferation; CC: cell cycle; BS: brain segmentation; LM: lipid metabolism; ST: signal transduction; M: metabolism; CM: cell migration, D: differentiation; CG: cell growth; A: apoptosis; BAB: bile acid biosynthesis; XM: xenobiotic metabolism; T: transport; IM: inactivation of MAPK; R: receptor; ANG: angiogenesis.

In normal rat liver parenchyma of rats treated with 5000 ppm Valerian after vehicle administration, only expression of Gsta2 and adenylate kinase 3 (Ak3) was significantly increased.

### Gene clustering and comparison analysis of upstream regulators by IPA

**Figure 3:**
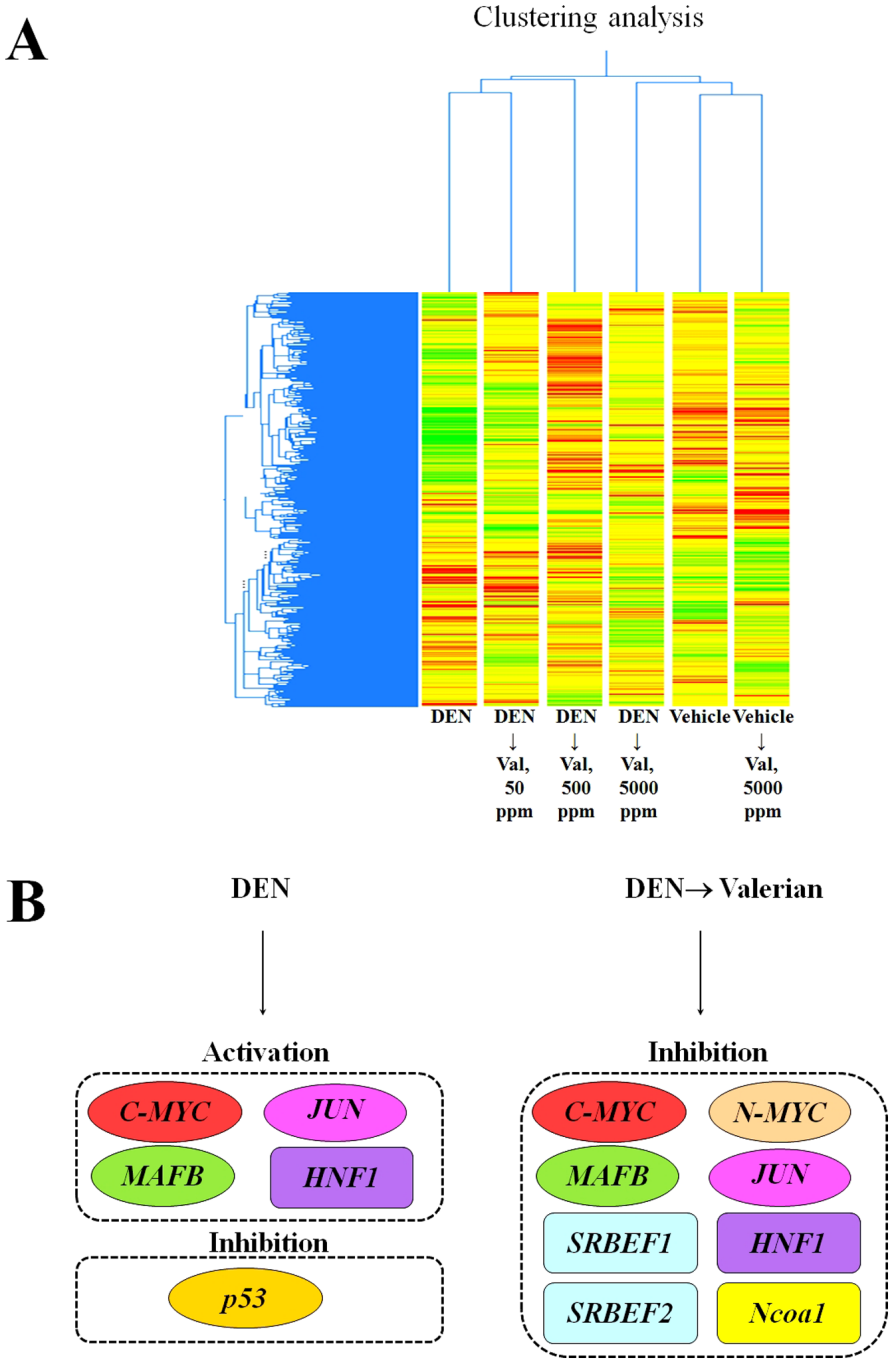
Gene Spring Clustering analysis of Affimetrix cDNA microarray data (A) and summary of comparative analysis of upstream regulators for which activation of inhibition was predicted by IPA analysis concerning liver tumorigenesis in rats given DEN or DEN followed by 50, 500 and 5000 ppm Valerian (B).

Clustering analysis by Gene Spring software demonstrated clear differences in gene expression pattern between vehicle and DEN treated groups (Fig.3A). Valerian application after DEN initiation restored expression of numerous genes, returning it to normal. Thus, gene expression pattern in DEN→5000 ppm Valerian group was similar to that of vehicle and vehicle→5000 ppm Valerian groups (Fig.3A). However, those of DEN initiation group was the most close to DEN followed by 50 ppm Valerian group.

IPA upstream regulator analysis indicated that DEN treatment resulted in activation of c-myc, Mafb, jun and HNF1 (Fig.3B). In contrast, inhibition of c-myc and Mafb, N-myc, Jun, SRBEF1/2, hepatocyte nuclear factor 1 (HNF1) and nuclear receptor coactivator 1 (Ncoa1) upstream regulators in all Valerian treated groups (z-score ≥2.0) were predicted by IPA (Fig.3B).

### Alteration in mRNA expression of genes involved in GABA signaling, apoptosis, cell proliferation and formation of oxidative stress

To investigate mRNA expression of other genes involved in GABA(A)RA1 signaling and other intracellular pathways, and to confirm the results of cDNA microarray analysis, GABA(A)RA1, HDAC4, c-myc, Mafb, jun, fos, CD1, NfkB, ERK1, p38, Nrf2, NQO1, HO-1, Gpx2, SOD, CAT and CYP7A1, p53, p21^Waf1/cip1^ and Bax, was checked by Q-RT-PCR analysis (Table 4). Significant coordinated elevation of GABA(A)RA1 and its upstream regulator, HDAC4, was found in Valerian treated rat livers after the DEN initiation as compared to DEN control group. Furthermore, significant increase of mRNA levels in initiation control and dose-dependent inhibition of c-myc, Mafb, CD1 and CYP7A1 expression in Valerian-treated groups was noted. Interestingly, suppression of famous indicator of oxidative stress and GABA(A)RA1-related transcriptional factor, Nrf2 and its downstream genes NQO1 and Gpx2 was obvious (Table 4). In addition, we observed significant dose-dependent induction of CAT expression, but not HO-1 or SOD by Valerian as compared to DEN initiation control (Table 4). On the contrary, no changes in expression of genes involved in MAPK signaling (ERK1, p38) or NfkB were found (data not shown). Furthermore, expression of genes regulating apoptosis, such as p53, Bax and p21^Waf1/Cip1^ was suppressed in DEN-treated animals but induced dose-dependently by Valerian treatment (Table 4).

**Table 4:** Alterations of mRNA expression of genes involved in GABA signalling, formation of oxidative stress, apoptosis and cell proliferation induced by administration of Valerian at different doses detected by real-time Q-RT-PCR.

	DEN	DEN→Val, 50 ppm	DEN→Val, 500 ppm	DEN→Val, 5000 ppm	Vehicle	Vehicle→Val, 5000 ppm
**GABA signaling**						
GABA(A)RA1	0.59±0.36	0.59±0.27	1.18±0.57^a^	1.18±0.51^a^	0.57±0.34	0.72±0.27
HDAC4	0.98±0.21	1.11±0.25	1.12±0.29	1.52±0.45^a^	1.72±0.43^b^	1.68±0.54
**Oxidative stress**						
Nrf2	0.74±0.15	0.34±0.09^b^	0.38±0.13^a^	0.37±0.09^a^	0.68±0.25	0.66±0.22
NQO1	0.98±0.30	0.63±0.15^a^	0.62±0.21^a^	0.60±0.29^a^	0.63±0.29^a^	0.64±0.32
Gpx2	1.23±0.38	0.82±0.27	0.73±0.14^b^	0.68±0.25^a^	1.04±0.33	1.11±0.61
CYP7A1	26.52±13.55	11.83±7.31^a^	6.96±2.96^b^	4.26±2.38^b^	1.60±0.84	1.66±0.55
HO-1	0.87±0.32	0.74±0.10	0.81±0.15	0.92±0.12	1.19±0.31	1.08±0.34
SOD	1.01±0.53	0.87±0.15	0.9±0.21	0.92±0.18	1.22±0.32	1.19±0.43
CAT	0.79±0.24	0.81±0.20	1.18±0.27^a^	1.60±0.65^a^	1.77±0.56^b^	1.78±0.80
**Apoptosis**						
p21^Waf1/Cip1^	1.52±0.43	1.81±0.61	2.23±0.75^a^	2.20±0.36^a^	1.00±0.37	1.25±0.37
p53	0.65±0.11	0.63±0.10	0.68±0.10	0.95±0.28^a^	0.83±0.18	0.91±0.18
Bax	0.51±0.10	0.55±0.09	0.58±0.08	0.87±0.26^a^	0.78±0.25	0.79±0.30
**Cell proliferation**						
c-myc	8.25±2.28	4.60±2.92	1.79±0.89^c^	1.68±1.01^c^	2.83±1.88^b^	1.40±0.73
MafB	5.93±3.23	5.86±3.10	3.10±1.53	1.14±0.94^b^	1.58±0.79^b^	1.80±0.96
jun	0.65±0.28	0.59±0.21	0.47±0.19	0.54±0.17	0.69±0.16	0.80±0.29
CD1	2.61±0.87	1.34±0.37^b^	1.10±0.29^b^	1.22±0.27^b^	1.48±0.71^a^	1.17±0.47

The results are Mean ± SD. ^a^P<0.05; ^b^P<0.01 and ^c^P<0.001 vs DEN group. HDAC4, histone deacetylase 4; NQO1, NADPH quinone oxidoreductase; Gpx2, glutathione peroxidise 2; HO-1, heme oxegenase 1; SOD, superoxide dismutase; CAT, catalase; CD1, cyclin D1.

## Discussion

The present study demonstrated an inhibitory effect of Valerian on formation of GST-P^+^ foci in a medium-term rat liver bioassay indicating prevention of hepatocarcinogenesis. Importantly, clear dose dependent effects were obvious, and significant inhibition was observed even at low dose. The mechanisms are likely to be related to significant suppression of cell proliferation and induction of apoptosis in the areas of GST-P^+^ foci accompanied by inhibited formation of oxidative base modifications in the rat liver DNA, due to activation of GABA(A)R-mediated signaling, coordinated with induction of HDAC4 and GABA(A)RA1, CAT, p53, p21^Waf1/cip1^ and Bax, and inhibition of c-myc, Mafb, CD1, CYP7A1 and Nrf2.

In this study, Valerian was also found to suppress the serum levels of AST, a pyridoxal phosphate-dependent transaminase enzyme which was induced by DEN treatment. AST is usually found in the liver, heart, skeletal muscle, kidneys, brain, and red blood cells, and it is commonly measured clinically as a marker for liver health. In line with our data, previously AST elevation in the rat blood serum and its suppression by potential chemopreventive agents was shown after DEN injection in rats and mice [17], being indicative of liver damage induced by this genotoxic hepatocarcinogen.

Here we present the first evidence for inhibition of cell proliferation with Valerian in the areas of GST-P^+^ foci. Interestingly, in the present study positive expression of GABA(A)RA1 (α1 subunit) with a membranous and/or cytoplasmic localization was found in cells comprising GST-P^+^ foci in rats initiated with DEN. Furthermore, Valerian administration caused GABA(A)RA1 elevation in GST-P^+^ foci. GABA(A)R is an ionotropic receptor and ligand-gated ion channel and upon activation, exhibits chloride channel activity selectively and conducts Cl^−^ through pores resulting in hyperpolarization of the neuron. This causes an inhibitory effect on neurotransmission by diminishing the chance of a successful action potential occurring. To date, 16 human and rat GABA(A)R subunit genes have been characterized and grouped together according to their amino acid similarity and termed: α1-6, β1-3, γ1-3, δ, ε, θ, and π [18]. In the brain, GABA(A)Rs are thought to be composed of 2 α, 2 β subunits and one other such as γ or δ subunit and the potency of GABA(A)R agonists is influenced by the subunit composition [19]. The major subtype of GABA(A)R, α1-containing benzodiazepine (BzD) receptor site, have been proposed to be responsible for the sedative action; the α2 and/or the α3 subtypes have been suggested to mediate the anxiolytic activity and the myorelaxation effects, and the α5 subtype has been associated with cognition processes [20]. Whereas GABA acts at the two extracellular β (+) α (−) interfaces of GABA(A)Rs, the allosteric modulatory benzodiazepines interact with the extracellular α (+) γ2 (−) interface [21]. The γ2-subunits of GABA(A)Rs combine with α1-3,5-subunits to form receptors that are sensitive to benzodiazepines [19].

For comparison, GABA(B) receptors (GABA(B)R) are metabotropic transmembrane receptors for GABA that are linked via G-proteins to potassium channels [22]. The changing potassium concentrations hyperpolarize the cell at the end of an action potential. GABA(B)Rs are similar in structure to and in the same receptor family with metabotropic glutamate receptors and can reduce the activity of adenylyl cyclase and decrease the cell's conductance to Ca^2+^ [23]. Previously, differential effects of GABA(A)R and GABA(B)R agonists on rat liver have been demonstrated [6],[7]. Thus, GABA(A)R but not GABA(B)R was shown to act as an inhibitory signal for hepatic cell proliferation. In these studies, a GABA(A)R agonist, muscimol, inhibited epidermal growth factor (EGF) induced DNA synthesis and enhanced the transforming growth factor β1 (TGFβ1) mediated DNA synthesis suppression in primary hepatocyte cultures. Importantly, GABA(B)R enhancement induced hepatic neoplasia [7]. Its agonist, baclofen, exerted completely opposite effects to muscimol in primary hepatocyte cultures acting as a potent co-mitogen, triggering DNA synthesis mediated through G(i) protein coupled GABA(B)Rs [7]. GABA content was further shown to be decreased in brain stems of PH and DEN-treated rats. GABA(A)R number and affinity in brain stem membrane preparations of these rats were significantly decreased, but GABA(B)R number and affinity were increased [8]. Moreover, it has been recently shown that autocrine/paracrine GABA signaling by means of GABA(A)Rs negatively controls embryonic stem and peripheral neural crest stem cell proliferation, as well as preimplantation embryonic growth and proliferation in the boundary-cap stem cell niche, resulting in attenuated generation of neuronal progenies [9]. Activation of GABA(A)Rs was suggested to lead to hyperpolarization, increased cell volume and accumulation of stem cells in S phase, thereby causing a rapid decrease in cell proliferation. The signaling pathway involved GABA(A)Rs with signals through S-phase checkpoint kinases of the phosphatidylinositol-3-OH kinase-related kinase family and the histone variant H2AX, thereby critically regulating stem cell proliferation [9]. Furthermore, GABA itself was reported to regulate the proliferation and growth of embryonic and neural progenitor cells [24], in addition to their migration [25] and differentiation [26]. Thus, inhibition of rat liver cell proliferation by Valerian after DEN treatment and PH observed in our study could be due to (i) direct effects of Valerian on the rat liver GST-P^+^ foci or (ii) indirect influence on GABAergic neurotransmission and GABA(A)R signaling in the CNS which inhibits hepatic proliferation via suppression of sympathetic regulation (Figure S2). Interestingly, overall increase of GABA(A)R activity was further shown to inhibit proliferation of the HepG2 human hepatocellular carcinoma cell line [27]. In light of these findings, the fact that GST-P^+^ foci overexpress GABA(A)RA1 allows us to suggest that Valerian may directly affect the cells comprising GST-P^+^ foci, thus, activating GABA(A)Rs, suppressing cell proliferation and finally exhibiting inhibitory effects on hepatocarcinogenesis.

Valeriana *sitchensis*, a native of northwestern America, is considered to have higher levels of valepotriates and stronger medicinal activity than other Valerian species but to contain only traces of valerenic acid [11],[28]. Its chemical components include numerous iridoid valepotriates, constituents of volatile oil (valeric and isovaleric acids, bornyl acetate, monoterpens), glycosides, alkaloids, free amino acids such as GABA, alanine, arginine and glutamine, camphene, manganese, calcium and others. Research into physiologic activity of Valerian individual components has demonstrated sedative effects [29],[30]. Valepotriates were first isolated in 1966 and contribute to the overall Valerian activity by possessing sedative effect on the CNS although their mode of action is not clearly established. They have been considered as a new class of cytotoxic and antitumor agents, however, being unstable, they act as prodrugs transformed into homobaldrinal [31]. Most of them contain one or two isovalerate moieties in the molecules and their decomposition has potential of yielding the isovaleric acid, which might be also responsible for their pharmacological activity [32]. The valepotriates (mostly valtrates) were reported to have some affinity for BzD sites in peripheral GABA(A)Rs, which differ from those found in the CNS and are located mainly in peripheral tissues and glial cells in the brain, and the barbiturate receptors to promote inhibition of degradation of GABA [33],[34]. Valeric and mostly isovaleric acids were demonstrated to bind GABA(A) and glycine receptors, however, the distinct mechanisms of action remain unclear [30],[32]. The effect of well-studied valerenic acid, which is found in the present extract only in trace amounts, is selective for GABA(A)Rs containing β2 and/or β3 subunits [35]. Importantly, decreased levels of GABA(A)R-β3 were observed in human hepatocellular carcinoma, while α3 was suggested to play an opposite role [27]. Valerian root extracts also contain some amounts of GABA which could directly cause sedation but there is some controversy surrounding the bioavailability of this compound [29]. Importantly, GABA itself has been shown to be an immunomodulator and to exert antitumorigenic activity *in vitro* and *in vivo* on lung, pancreas, colon, breast and prostate cancers [27],[36],[37]. Although many components are believed to be responsible for Valerian biologic effects, it is likely that all of the active constituents act in a synergistic manner to produce a clinical response [29].

The selected Valerian doses in this study were comparable to those applied in humans if using the extrapolation with multiplication index for rats [12] or the extrapolation to a human equivalent dose (HED) with the body surface area (BSA) normalization method (mg/m^2^ conversion) [38]. Thus, in previous placebo-controlled trials, adults were administered Valerian extract (400–600 mg/day, equal to 8–12 mg/kg b.w./day intake for an individual with a mean body weight of 50 kg) for significant improvement in sleep quality and daytime mood [1],[39]. In another randomized double-blind study the effects of low doses of 60 mg/day (1.2 mg/kg b.w./day) and 120 mg/day (2.4 mg/kg b.w./day) Valerian were investigated in adults to detect improvement of insomnia, and 120 mg/day was decided as an effective dose [40].

As detected by cDNA microarray analysis, Valerian treatment at all doses suppressed expression of numerous genes affecting cellular proliferation, such as *c-myc*, *Mafb* oncogenes, *Per2*, *Nr0b2*, *Igfbp1*, *CD1* and others. Furthermore, it inhibited *N-myc* and *jun* oncogenes as indicated by the analysis of upstream regulators by IPA (Figure S2). These alterations may explain its inhibitory activity on cell proliferation in GST-P^+^ foci and normal-appearing liver tissue. Furthermore, Valerian application induced elevation of mRNA expression of genes inducing apoptosis such as *p21^WAF1/Cip1^*, *p53, BAX* and *Itpr1*. In line with our data, previously, induction of apoptosis by sedative chemicals has been explained on the basis of its ability to activate *p53* [41] and *p21* ^WAF1*/*Cip1^ [42] gene expression. It is conceivable that observed alterations of mRNA expression of *c-myc*, *mafb* and other genes controlling cell proliferation and possibly apoptosis are likely to be mediated by GABA(A)R signaling.

GABA(A)RA1 expression was reported to be positively regulated by HDAC4 in cultured neurons [43]. In the present study, we observed significant increase in GABA(A)RA1 mRNA and protein expression which was coordinated with HDAC4 overexpression in the liver of rats administered Valerian. Thus, GABA(A)RA1 is likely to be controlled by HDAC4. Furthermore, suppression of another GABA(A)RA1-related transcriptional factor, Nrf2, and its downstream genes, NQO1 and Gpx2 expression in the liver of rats treated with Valerian suggested that Valerian could suppress the formation of oxidative stress in the rat liver by inhibiting the Nrf2 signaling pathway, which could be GABA(A)RA1-dependent (Figure S2) [44],[45]. We further confirmed inhibition of Nrf2 immunohistochemically demonstrating suppression of Nrf2-Ser-P expression in the livers of Valerian treated rats.

8-OHdG, the most sensitive and useful marker of oxidative DNA adducts, is known to be produced by exposure to various carcinogens and to cause mutations [14]. Significant increase of 8-OHdG levels in the DEN initiation group over the vehicle controls associated with rise of GST-P^+^ foci observed in the present study supported this concept. Therefore, the suppression of their development by Valerian might be related to an inhibitory effect on 8-OHdG formation in the DNA of hepatocytes. The observed suppression of 8-OHdG generation by Valerian after DEN initiation might be a result of suppression of oxidative stress due to up-regulation of catalase (*CAT*), down-regulation of *Nrf2* as well as *CYP7A1* in the rat liver (Figure S2).

In conclusion, Valerian, a sedative, hypnotic and anxiolytic medicine, here inhibited the formation of GST-P^+^ foci by activating GABA(A)R-mediated signaling in rats. Our data demonstrate that Valerian suppressed 8-OHdG formation, significantly inhibited cell proliferation and induced apoptosis in the areas of GST-P^+^ foci, and altered expression of genes related to control of cell proliferation and apoptosis, which might explain its inhibitory effects on hepatocarcinogenesis.

## Supporting Information

Figure S1 **Immunohistochemistry for Nrf2-Ser-P in DEN initiation control (A, B), DEN followed by 5000 ppm Valerian (C, D), and Vehicle groups (E, F).** Note the intense Nrf2-Ser-P staining in the portal central vein areas in DEN control group, but absence of staining in Valerian-treated and Vehicle groups.
(TIF)

Figure S2 **Graphically illustrated suggested mechanisms of Valerian inhibitory activity on rat hepatocarcinogenesis.**
(TIF)
