# Supplementary figures and images for: Valerian Inhibits Rat Hepatocarcinogenesis by Activating GABA(A) Receptor-Mediated Signaling
Source: PLoS One. 2014 Nov 24;9(11):e113610. doi: 10.1371/journal.pone.0113610 (PMC4242630; doi:10.1371/journal.pone.0113610)

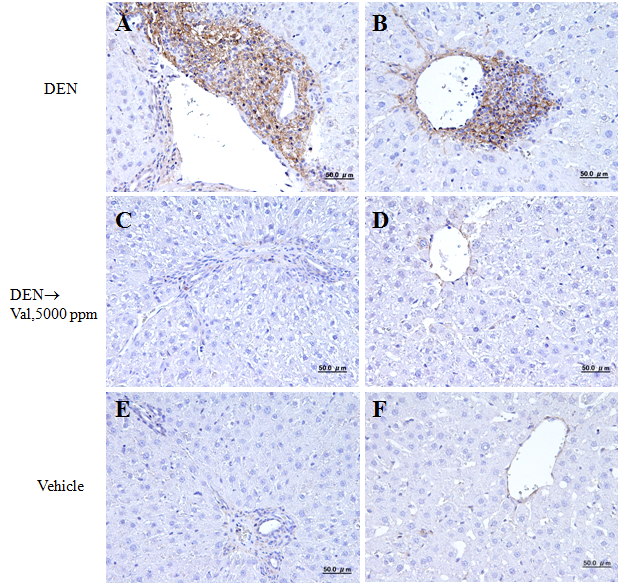

Supplement: Figure S1 — Immunohistochemistry for Nrf2-Ser-P in DEN initiation control (A, B), DEN followed by 5000 ppm Valerian (C, D), and Vehicle groups (E, F). Note the intense Nrf2-Ser-P staining in the portal central vein areas in DEN control group, but absence of staining in Valerian-treated and Vehicle groups. (TIF) [file pone.0113610.s001.tif]

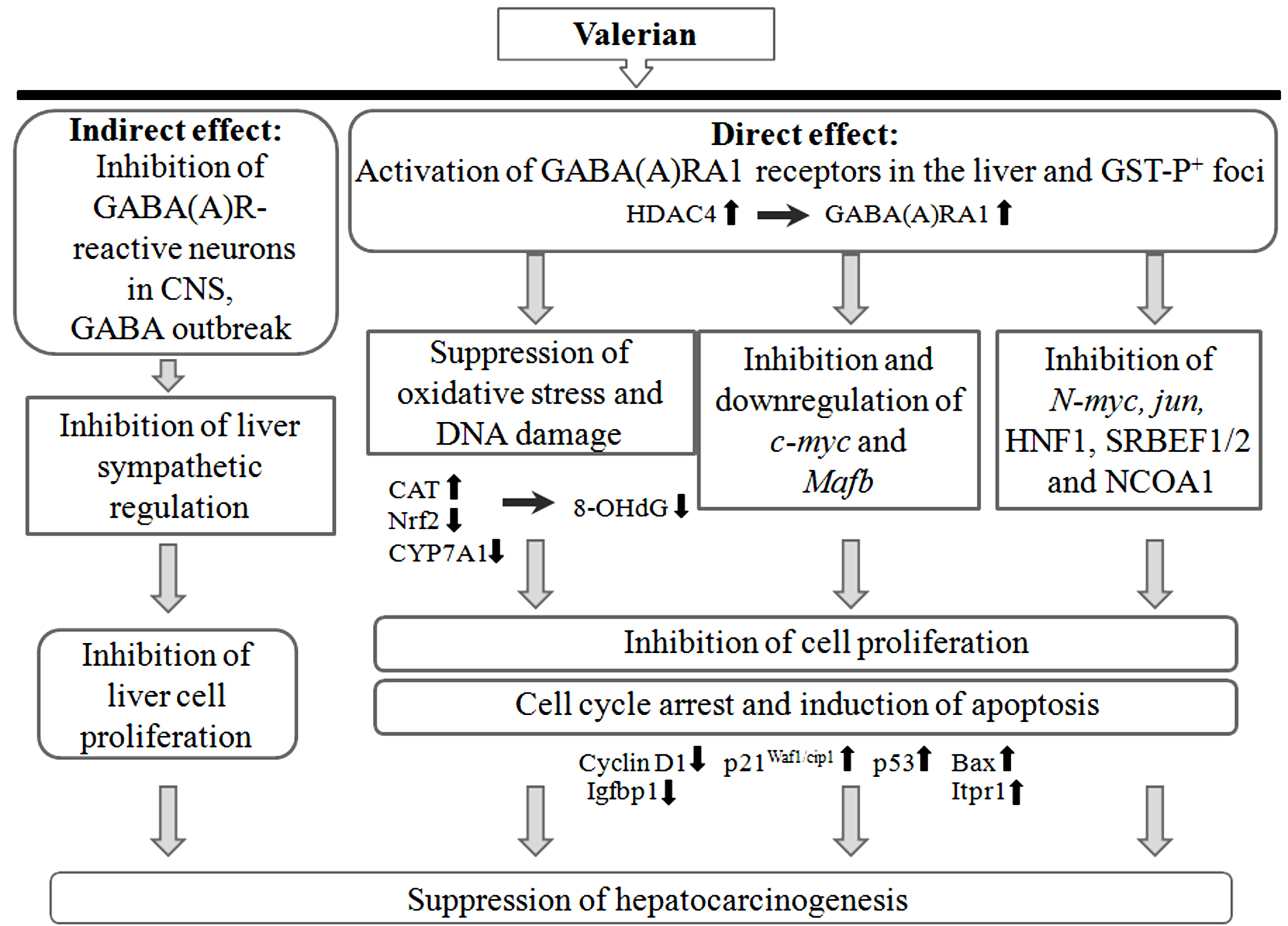

Supplement: Figure S2 — Graphically illustrated suggested mechanisms of Valerian inhibitory activity on rat hepatocarcinogenesis. (TIF) [file pone.0113610.s002.tif]
